# Cleats on, battle real: sustainability and gender equality in women’s football

**DOI:** 10.3389/fpsyg.2026.1743515

**Published:** 2026-03-23

**Authors:** Serda Ornek, Murat Yalcin Besiktas, Mustafa Serdar Terekli, Mert Erkan, Nalan Aksakal

**Affiliations:** 1 Faculty of Sport Sciences, Department of Sport Management, Fenerbahce University, Istanbul, Türkiye; 2 Faculty of Sport Sciences, Department of Coach Education, Fenerbahce University, Istanbul, Türkiye; 3 Department of Physical Education and Sports Teaching, Faculty of Sport Sciences, Fenerbahce University, Istanbul, Türkiye; 4 Faculty of Sport Sciences, Department of Physical Education, Eskisehir Technical University, Eskisehir, Türkiye

**Keywords:** gender equality, policy development, sport sustainability, structural barriers, women’s football

## Abstract

**Objective:**

This study aims to examine the rise of women’s football in Turkey and the challenges faced by female footballers both on and off the field from the perspectives of gender and sustainability.

**Methods:**

Conducted with a phenomenological design, the study involved semi-structured interviews with 18 female footballers playing in the Super League between June and September 2025, using a snowball sampling method. The data were analyzed thematically.

**Results:**

The findings revealed that the experiences of female footballers are shaped at the intersection of individual motivation, environmental support, and structural inequalities. Participants indicated that gender biases, economic constraints, and lack of media visibility limit their career development. Inadequate facilities, scarcity of female coaches, and limited professional opportunities were also identified as key barriers.

**Conclusion:**

Women’s football is not only a sporting activity but also a social arena with the potential to foster gender equality and sustainable development. The development of inclusive strategies by the Turkish Football Federation and clubs—such as equal pay policies, sponsorship support, female coach training programs, and “female-friendly” facility arrangements—is critically important for the sustainable growth of women’s football.

## 1 Introduction

Football has long been constructed as a masculine domain, shaped by hegemonic norms that associate physical strength, competitiveness, and endurance with male identity (Connell and Messerschmidt, 2005; Messner, 2002). Within this gendered sporting culture, women’s participation in football has frequently been framed as deviant or secondary, reinforcing structural and symbolic inequalities (Hargreaves, 1994; Pfister, 2015). Rather than being a neutral physical activity, football functions as a social institution in which gender hierarchies are reproduced, negotiated, and sometimes resisted.

Historically, women’s football has encountered institutional barriers across different national contexts. The 1921 ban imposed by the English Football Association remains a symbolic example of formal exclusion (Williams, 2003). Although explicit prohibitions have disappeared, structural inequalities persist in contemporary football systems through unequal funding, media representation, and professional opportunities (Agergaard and Tiesler, 2014; FIFA, 2023). In Turkey, women’s football began to institutionalize in the 1990s; however, disparities in economic resources, infrastructure, and public visibility continue to limit its development (Öztürk, 2019; Öztürk, 2019; Gültekin, 2021).

Media representations further reinforce these inequalities. Research consistently shows that female athletes are more likely to be portrayed through narratives emphasizing appearance, femininity, or personal life rather than athletic competence (Bruce, 2016; Toffoletti and Palmer, 2017). This pattern constrains the recognition of women’s football as a legitimate professional domain and shapes public perceptions of female athletic identity.

Beyond institutional and representational inequalities, women’s football must also be understood through its psychological dimensions. Participation in sport is not sustained solely by structural access but by the extent to which athletes experience meaningful engagement and psychological fulfillment (Ryan and Deci, 2017). Self-Determination Theory (SDT) provides a useful framework for understanding the motivational processes underlying sustained sport participation. According to SDT, optimal functioning depends on the satisfaction of three basic psychological needs: autonomy, competence, and relatedness (Ryan and Deci, 2020). When these needs are supported, athletes demonstrate higher well-being, resilience, and long-term commitment; when thwarted, motivation and career sustainability are undermined (Weinberg and Gould, 2019).

Recent sport psychology research highlights that gendered environments can systematically frustrate these psychological needs (Ntoumanis et al., 2021; Appleton and Duda, 2016). For female footballers operating within male-dominated structures, autonomy may be restricted by limited decision-making power, competence may be questioned through stereotypical narratives, and relatedness may be challenged by exclusionary team or institutional cultures. Consequently, sustainable participation in women’s football is closely tied to the broader sociocultural context in which athletes construct their identities.

In this regard, sustainability in sport should be conceptualized not only in economic or institutional terms but also in psychological terms—namely, the capacity of athletes to maintain motivation, well-being, and career continuity over time (Sarkar and Fletcher, 2014; Galli and Gonzalez, 2015). Examining women’s football through the integrated lenses of gender theory and SDT allows for a multidimensional understanding of how structural inequalities intersect with individual motivational processes. Accordingly, this study explores the lived experiences of female footballers in Turkey by addressing the following research questions: (1) How do female footballers experience structural and cultural barriers within football? (2) How do these conditions influence their psychological need satisfaction and career sustainability? By situating personal narratives within a theoretically grounded framework, the study seeks to contribute to discussions on gender equality, psychological well-being, and sustainable development in sport.

## 2 Materials and methods

### 2.1 Research design

This study adopted a qualitative research design to explore the lived experiences of female footballers within the broader sociocultural and institutional context of Turkish football. Qualitative inquiry enables an in-depth examination of how individuals interpret and make meaning of their experiences in socially constructed environments (Creswell and Poth, 2018). Given the study’s focus on gendered experiences and psychological sustainability, a phenomenological approach was employed to capture the essence of participants’ subjective realities.

Phenomenology seeks to understand how individuals experience and interpret a particular phenomenon in their everyday lives (Moustakas, 1994). Rather than aiming for generalization, this approach emphasizes depth, meaning, and contextual understanding. In the present study, the phenomenological framework allowed for the examination of how female footballers negotiate identity, structural inequality, and professional aspirations within a male-dominated sporting culture.

While the study was not designed as a deductive test of Self-Determination Theory (SDT), the theory informed the analytical lens as a sensitizing framework (Ryan and Deci, 2017). In particular, attention was paid to how participants described experiences related to autonomy, competence, and relatedness within their sporting environments. This theoretically informed but inductive orientation enabled the findings to remain grounded in participants’ narratives while allowing connections to established motivational theory.

### 2.2 Participants

A purposive sampling strategy was employed to recruit participants who could provide rich and relevant insights into the phenomenon under investigation (Patton, 2015). Specifically, criterion-based purposive sampling was used, with the primary inclusion criterion being active participation in the Turkcell Women’s Football Super League during the 2024–2025 season. Given the relatively limited accessibility of elite female footballers, snowball sampling was also utilized to reach additional participants through professional networks.

Eighteen female footballers participated in the study. The sample was considered sufficient to reach thematic depth and information power, as participants shared comparable professional contexts while providing diverse personal backgrounds (Malterud et al., 2016). Participants ranged in age from 20 to 32 years and represented multiple clubs within the league. For reporting purposes and to enhance participant anonymity, ages were grouped into three categories (20–24, 25–29, and 30–34), as presented in Table 1. To ensure confidentiality, participants were assigned codes (P1–P18), and identifying information was removed during transcription and reporting.

**Table 1:** Participant characteristics.

Participant	Age	Education level	Mother’s education	Mother’s occupation	Father’s education	Father’s occupation	Number of siblings	Club
P1	32	University	Primary	Homemaker	Primary	Military	9	Yüksekova
P2	22	University	Not reported	Homemaker	Not reported	Furniture craftsman	2	Fenerbahçe
P3	23	University	Not reported	Tailor	Not reported	Security	2	Fenerbahçe
P4	20	University	Not reported	Homemaker	Not reported	Security	5	Fenerbahçe
P5	26	University	High school	Homemaker	Primary	Textile worker	2	Yüksekova
P6	23	University	Secondary	Homemaker	Secondary	Tradesman	6	Trabzonspor
P7	24	University	Primary	Homemaker	Middle school	Deceased	2	Amed
P8	25	University	Primary	Homemaker	Primary	Deceased	3	Fatih Vatan Spor
P9	27	University	Primary	Homemaker	Middle school	Worker	Not reported	Galatasaray
P10	32	University	Primary	Homemaker	Middle school	Self-employed	2	Fomget
P11	25	University	Primary	Homemaker	Middle school	Self-employed	3	Fomget
P12	31	Master’s	Bachelor’s	Homemaker	Bachelor’s	Retired teacher	2	Beşiktaş
P13	27	University	High school	Homemaker	High school	Civil Servant	2	Beşiktaş
P14	31	University	Primary	Homemaker	Primary	Tradesman	4	Galatasaray
P15	25	University	High school	Homemaker	High school	Retired worker	2	Beşiktaş
P16	27	University	Primary	Homemaker	Middle school	Self-employed	1	Fenerbahçe
P17	28	University	Middle school	Homemaker	Primary	Tradesman	3	Trabzonspor
P18	21	University	Primary	Homemaker	Middle school	Civil servant	2	Trabzonspor

### 2.3 Data collection

Data were collected between June and September 2025 through semi-structured individual interviews. Semi-structured interviews provide flexibility while maintaining alignment with research objectives, allowing participants to elaborate on issues they consider meaningful (Creswell and Poth, 2018).

Interview questions focused on career trajectories, experiences of gender-related challenges, institutional support mechanisms, identity negotiation, and motivational processes. Each interview lasted approximately 30–35 min. When face-to-face meetings were not feasible, interviews were conducted via secure online video conferencing platforms.

All participants provided informed consent prior to participation. Interviews were audio-recorded with permission and transcribed verbatim. Participation was voluntary, and participants were informed of their right to withdraw at any stage without consequence.

### 2.4 Data analysis

Data were analyzed using reflexive thematic analysis as conceptualized by Braun and Clarke (2019, 2021). Reflexive thematic analysis emphasizes the active role of the researcher in knowledge production and acknowledges that themes do not passively emerge from data but are generated through interpretative engagement.

The analysis followed six recursive phases: familiarization with the data, initial coding, theme construction, theme review, theme definition, and reporting (Braun and Clarke, 2019). Rather than relying on a rigid coding frame, coding remained flexible and iterative. The researcher maintained reflexive notes throughout the process to critically examine interpretative assumptions and positionality.

Although the analysis was primarily inductive, the interpretation of themes was informed by SDT as a theoretical lens, particularly in relation to participants’ accounts of autonomy support or frustration, competence validation or undermining, and experiences of belonging or exclusion (Ryan and Deci, 2020).

The final analysis generated three overarching themes:

1. Career development and professional sustainability
2. Identity negotiation and gendered experience
3. Structural inequality and institutional constraints

These themes reflect both participants’ lived realities and the broader socio-psychological framework guiding the study.

## 3 Findings

### 3.1 Career and professional journey

Participants’ early engagement with football largely occurred through informal, male-dominated street play environments. Most described beginning to play football in childhood, typically alongside boys in their neighborhoods. This early exposure reflects how access to football for girls was not structured through institutional pathways but rather negotiated within gendered social spaces. For many, these informal beginnings became the foundation for developing a sense of competence, as they gradually recognized their skills and potential in comparison with male peers. As one participant noted, playing with boys from primary school allowed her to “realize her talent” and eventually transition into organized club football.

The transition from informal play to organized sport was often facilitated by physical education teachers, who served as critical gatekeepers in identifying and legitimizing participants’ abilities. Teachers not only recognized talent but also provided structural access to clubs. In this sense, they played a crucial role in supporting athletes’ emerging competence and enabling autonomy in pursuing football as a career. Without such institutional encouragement, several participants suggested that their engagement in football might not have progressed beyond recreational play.

Family support emerged as a decisive factor shaping athletes’ professional trajectories. While approximately half of the participants described receiving strong familial encouragement, others reported resistance rooted in conservative gender norms. In these cases, pursuing football required negotiating deeply embedded beliefs about femininity and appropriate gender roles. Participants who faced opposition often described their journeys as involving emotional struggle and social risk. From a psychological perspective, limited family support constrained the need for relatedness, whereas supportive families enhanced emotional security and sustained motivation. Even when initial resistance was present, eventual family acceptance often became a powerful source of validation.

Economic conditions further shaped professional experiences. Although most participants reported earning income from football, they emphasized that financial compensation remained insufficient, particularly in comparison to male footballers. The persistent income disparity reinforced a broader perception of structural inequality within the sport. Financial precarity sometimes required athletes to seek additional employment, which potentially undermined their sense of professional legitimacy and autonomy. While football provided identity and purpose, economic instability complicated the sustainability of their careers.

Balancing professional identity with personal life produced varied experiences. A majority of participants reported successfully separating on-field aggressiveness from their private selves, suggesting a capacity to maintain psychological autonomy across social contexts. However, others described reduced social time, emotional fatigue, and the difficulty of being physically distant from family members. These accounts reveal the complex negotiation between professional commitment and personal relatedness. The demands of elite sport required discipline and sacrifice, occasionally narrowing the space for social relationships beyond the team environment.

Overall, participants’ career trajectories illustrate how competence development, autonomy in career choice, and relational support dynamically interact within a gendered sporting structure. While many athletes demonstrated strong intrinsic commitment to football, their professional journeys were continuously shaped by structural, familial, and economic constraints.

### 3.2 Identity, gender, and being a woman in football

Participants overwhelmingly rejected the characterization of football as a “male sport,” positioning themselves as legitimate and equal actors within the field. While acknowledging that football has historically been embedded within patriarchal structures, they emphasized that athletic performance and competitive commitment are not gender-bound. This rejection can be understood as an assertion of autonomy—a refusal to internalize socially imposed gender norms that define certain sports as inherently masculine. By claiming space within football, participants actively reconstructed gender boundaries rather than passively accepting them.

Despite this resistance, participants consistently reported that broader society lacks awareness of women’s football. Many described repeatedly having to explain their profession, clarify misconceptions, or justify their presence in the sport. This continual need for self-explanation was described as emotionally exhausting. From a Self-Determination Theory perspective, such societal misrecognition may undermine both competence and relatedness needs. When athletes’ professional identities are questioned, their legitimacy as skilled performers becomes socially fragile, potentially weakening perceived competence. Simultaneously, insufficient societal recognition limits their sense of belonging within the broader sporting culture.

Tensions between feminine identity and footballer identity further illustrate this negotiation. Several participants described moments of internal conflict, particularly regarding perceptions of aggressiveness or toughness associated with football. Some indicated that characteristics valued on the field did not always align with expectations placed on women in private life. This reflects the psychological work required to maintain autonomy across social domains—separating athletic intensity from personal identity while navigating gendered expectations. Rather than indicating identity confusion, these tensions demonstrate the complex integration of multiple social roles within restrictive gender frameworks.

Experiences of sexist remarks, verbal abuse, and negative spectator behavior were reported by all participants. Although some perceived the intensity of such behaviors as lower than in men’s football, exposure to harassment was nonetheless normalized within their competitive environments. Such experiences represent direct threats to relatedness and competence. Being subjected to insults or gender-based derogatory language signals exclusion from the sporting community and questions athletes’ legitimacy. However, many participants described coping strategies centered on psychological focus and collective resilience, suggesting strong internal motivation and team-based support systems.

Team solidarity emerged as a critical protective factor. Most participants emphasized that being part of an all-female team fostered empathy, mutual understanding, and emotional support. Shared experiences of navigating gender barriers appeared to strengthen relational bonds. In SDT terms, this strong sense of relatedness functioned as a buffer against external discrimination. Nevertheless, some participants acknowledged occasional interpersonal tensions, noting that jealousy or internal competition could disrupt team cohesion. These accounts suggest that while gendered solidarity enhances belonging, relational dynamics remain complex and context-dependent.

Overall, participants’ narratives reveal an ongoing negotiation between structural gender norms and personal athletic identity. Their rejection of football as a male-exclusive domain reflects autonomy; their insistence on professional legitimacy reflects competence; and their reliance on team solidarity underscores the centrality of relatedness. Yet these psychological needs are continuously shaped—and at times constrained—by societal perceptions and gendered power structures within sport.

### 3.3 Social, institutional, and representational dimension

Participants consistently emphasized that women’s football remains structurally underrepresented within mainstream media. National broadcasts tend to focus primarily on international matches, finals, or high-profile clubs, while most league matches receive limited or no television coverage. This restricted visibility was perceived as one of the most significant barriers to the development of women’s football. From a Self-Determination Theory perspective, limited media exposure can undermine athletes’ sense of competence, as public recognition and validation are central to professional legitimacy. When performance is not widely visible, opportunities for sponsorship, fan engagement, and broader social acknowledgment remain constrained.

Although social media platforms and club-based digital channels have increased visibility to some extent, participants highlighted that online streaming cannot fully replace national television broadcasting. The difference in audience reach was described as substantial. While digital content contributes to incremental recognition, the absence of consistent mainstream coverage reinforces the perception that women’s football occupies a secondary position within the sporting hierarchy. This structural invisibility not only affects financial sustainability but also shapes athletes’ sense of belonging within the broader football community, thereby influencing relatedness at a societal level.

At the institutional level, many participants described supportive environments within their own clubs. Some even reported experiencing what they perceived as positive discrimination, particularly within major clubs that actively invest in women’s teams. Such institutional backing can be interpreted as autonomy-supportive, as it legitimizes athletes’ professional choices and provides structural encouragement. When clubs allocate resources, prioritize women’s teams, and promote them publicly, they reinforce players’ sense of competence and professional worth.

However, this support was described as uneven across the league. Outside major clubs, participants reported limited resources, insufficient organizational structures, and financial instability. Budget inequalities were frequently mentioned as a major obstacle. Many athletes noted that smaller clubs rely heavily on personal contributions from club presidents or local sponsors. Financial precarity forces some players to seek additional employment, thereby constraining their autonomy and reducing their capacity to fully commit to their athletic development. Structural inequalities, therefore, directly shape the sustainability of women’s professional careers.

Participants also discussed the limited presence of women in technical and managerial positions. The scarcity of female coaches, physiotherapists, and administrators was perceived as a significant gap within the institutional framework. Many athletes expressed that female staff members better understand their physical and psychological needs, particularly in areas related to gender-specific health and communication. In SDT terms, the absence of female representation within leadership roles may restrict relatedness and reduce the sense of shared understanding within the sporting environment. Increasing female participation in decision-making structures was seen as essential for improving communication and fostering inclusive policies.

Finally, participants described infrastructural inadequacies in stadiums and training facilities. Issues such as insufficient locker rooms, hygiene problems, lack of hot water, and the use of artificial turf not designed for elite-level performance were commonly mentioned. These structural shortcomings signal that women’s football is not prioritized in facility planning. Beyond practical inconvenience, such conditions affect athletes’ perceived competence and professional status. Facilities that are not designed with female players in mind implicitly communicate marginalization, reinforcing broader patterns of gender inequality within sport.

Taken together, these findings illustrate that women’s football operates within a complex institutional landscape characterized by partial support and persistent structural constraints. While some clubs provide autonomy-supportive and competence-enhancing environments, broader systemic inequalities in media representation, financial allocation, leadership representation, and infrastructure continue to shape athletes’ experiences. These institutional dynamics interact directly with psychological needs, demonstrating how structural conditions influence motivation, identity, and professional sustainability in women’s football.

## 4 Discussion

The findings of this study demonstrate that women’s engagement in football in Turkey is shaped by the interaction of societal gender norms, structural inequalities, and institutional arrangements. When interpreted through the lens of Self-Determination Theory (SDT), these dynamics can be understood as contextual conditions that either support or frustrate the basic psychological needs for autonomy, competence, and relatedness. Rather than representing isolated challenges, the experiences of female footballers reflect a broader gendered motivational climate embedded within Turkish football culture.

Entry into football was largely mediated through brothers, male peers, and physical education teachers. While such relationships function as enabling mechanisms, they also reveal that access to football remains embedded within male-dominated social networks. From an SDT perspective, these actors operate as critical agents of relatedness support, facilitating initial participation. Cin (2022) similarly identifies physical education teachers as influential figures directing girls toward football. However, the fact that participation often depends on male-linked pathways suggests that relatedness satisfaction is conditional rather than structurally guaranteed. Consistent with Pfister (2010) and Öztürk (2017), family approval and social capital function as gatekeeping mechanisms, shaping the internalization of sport participation. When family support is present, it likely enhances autonomous motivation; when resistance occurs, athletes may experience motivational tension shaped by external pressures.

Economic precarity emerged as a central structural constraint. Participants’ emphasis on insufficient earnings and persistent pay gaps reflects more than financial inequality; it represents a restriction on autonomy. According to SDT, autonomy involves experiencing volition and the capacity to make meaningful life choices. When women’s football does not provide a sustainable career pathway, athletes’ long-term agency is limited. This finding aligns with Cooky and Messner (2018) argument that female athletes are systematically economically undervalued and with Öztürk (2017) identification of a “lack of future” scenario in Turkey. FIFA (2020, 2022) further documents the global prioritization of men’s football budgets, reinforcing that autonomy frustration operates at both national and international levels. Thus, economic inequality functions as a structural regulator of motivation rather than a peripheral issue.

Participants’ efforts to balance professional identity with personal life reflect ongoing negotiation between gender norms and athletic commitment. Bruening and Dixon (2008) and Culvin (2021) emphasize that gender roles shape female athletes’ work–life balance, and the present findings extend this by suggesting that such negotiations influence the stability of autonomous motivation. When societal expectations conflict with athletic identity, athletes must continually reconcile competing role demands. This dynamic reflects the tension between competence pursuit and culturally prescribed femininity.

Gender-based perceptions of football further illuminate how competence and relatedness are socially mediated. Although most participants rejected the idea that football is inherently a “male sport,” they reported being required to explain or defend their professional identities. This resonates with Pfister (2010) concept of social invisibility and Cooky and Messner (2018) discussion of symbolic marginalization. From an SDT perspective, competence involves not only skill acquisition but also social recognition. When female athletes’ legitimacy is questioned, competence validation becomes unstable. Similarly, Scraton and Flintoff (2013) observations regarding complex team dynamics highlight how solidarity and competition coexist, shaping relatedness satisfaction within gendered environments.

All participants reported exposure to sexist remarks and harassment, underscoring the persistence of male-dominated football culture (Gubby and Martin, 2024). Cleland et al. (2020) and Pope (2011) argue that football spaces remain deeply masculinized, and the Turkish context reflects this continuity (Koca and Bulgu, 2005; Öztürk, 2017). Within SDT, such experiences can be interpreted as relatedness frustration, where belonging becomes conditional. Yet participants’ coping strategies—such as focusing on gameplay—demonstrate psychological resilience, echoing Fielding-Lloyd and Meân (2011) emphasis on toughness among female athletes. This coexistence of structural exclusion and personal resilience suggests that motivational persistence often depends on individual coping rather than systemic support.

Media underrepresentation further reinforces competence and autonomy constraints. Limited broadcasting of women’s matches restricts public recognition and sponsorship opportunities. Turkish literature consistently frames women’s football as marginalized (Koca and Bulgu, 2005; Arık, 2003; Kavasoğlu, 2021), while international research documents gendered media framing (Cooky et al., 2015; Fink, 2015). Although social media provides alternative visibility (Öztürk and Koca, 2018), it does not substitute for institutional recognition. From an SDT standpoint, symbolic visibility contributes to competence affirmation; its absence reinforces marginalization.

Institutional dynamics reveal a more nuanced picture. Many participants described supportive club environments, particularly within major clubs such as Fenerbahçe, Galatasaray, and Beşiktaş. As Koca and, Bozlu (2021), and Öztürk and Koca (2018, 2022) note, investment by major clubs enhances visibility and resources. However, support remains uneven, reflecting what may be conceptualized as institutional inequality. While certain contexts foster autonomy and competence, systemic transformation across the league has not yet occurred. FIFA (2020, 2022) similarly indicates that institutional prioritization of men’s football persists globally.

The limited representation of women in technical roles introduces additional implications for motivational climate. Participants reported communication gaps with male coaches and highlighted the motivational benefits of female technical staff. LaVoi (2016) identify global structural barriers limiting women’s leadership in sport, while Bozlu (2021) documents similar patterns in Turkey. Within SDT, autonomy-supportive interpersonal environments are critical for sustained engagement. Zarrett et al. (2019) and Fasting and Pfister (2000) suggest that female coaches can enhance belonging and confidence, reinforcing relatedness and competence satisfaction. Therefore, gender imbalance in leadership positions affects not only equity but also psychological climate.

Finally, facility conditions reflect material manifestations of gender hierarchy. Inadequate locker rooms, hygiene problems, and male-oriented infrastructure illustrate how competence development is structurally constrained. Öztürk (2017) and Bozlu (2021) highlight similar disparities in Turkey, while Scraton and Flintoff (2013) demonstrate that gendered sports spaces indirectly limit participation. From an SDT perspective, environments that fail to provide safe and adequate resources undermine competence and relatedness simultaneously.

Overall, the findings suggest that women’s football in Turkey operates within a gendered motivational ecology in which autonomy, competence, and relatedness are unevenly supported. While individual resilience enables continued participation, sustainable development requires structural reforms that move beyond reliance on personal coping strategies. Economic investment, equitable infrastructure, expanded media representation, and increased female leadership are not only matters of equality but also essential conditions for fostering stable and autonomous motivation within women’s football.

## 5 Conclusion

This study examined the experiences of female footballers in Turkey through the lens of Self-Determination Theory, demonstrating that participation in women’s football is shaped by the uneven satisfaction of autonomy, competence, and relatedness within a gendered sport system. Rather than reflecting isolated individual challenges, the findings reveal how structural inequalities, cultural norms, and institutional arrangements collectively regulate motivational processes in women’s football.

The persistence of economic precarity, infrastructural inequality, limited media visibility, and gender imbalance in leadership positions indicates that the development of women’s football cannot be understood solely in terms of participation growth. From an SDT perspective, sustainable professionalization requires environments that consistently support athletes’ autonomy through viable career pathways, reinforce competence through equitable investment and recognition, and foster relatedness through inclusive and psychologically safe sporting climates.

Although signs of progress are evident—particularly in the increased involvement of major clubs—the overall ecosystem remains uneven. Consequently, women’s continued participation often relies on personal resilience rather than structurally supported motivation. Long-term advancement, therefore, depends on systemic reforms rather than individual coping strategies.

For governing bodies such as the Turkish Football Federation (TFF) and affiliated clubs, this implies the need for coordinated and gender-sensitive policies. Strategic investment in equitable facilities, transparent budget allocation, expanded media representation, and structured development pathways for female coaches are not only matters of fairness but essential mechanisms for enhancing motivational sustainability. Policies that strengthen both professional infrastructure and social support systems will contribute to environments in which female athletes can internalize their athletic identities without persistent negotiation of legitimacy.

Ultimately, the advancement of women’s football in Turkey requires a shift from symbolic inclusion to structural transformation. By addressing the interconnected motivational, institutional, and cultural dimensions identified in this study, stakeholders can foster a football environment that supports psychological need satisfaction and promotes both athletic excellence and gender equality. In doing so, women’s football may function not only as a competitive sport but as a meaningful site of social change.

## Data Availability

The raw data supporting the conclusions of this article will be made available by the authors, without undue reservation.

## References

[ref1] Agergaard S. Tiesler N. C. (2014). Women, Soccer and Transnational Migration. London: Routledge.

[ref2] Appleton P. R. Duda J. L. (2016). Examining the interactive effects of coach-created empowering and disempowering motivational climates on athletes’ health and functioning. Psychol. Sport Exerc. 26, 61–70. doi: 10.1016/j.psychsport.2016.06.007

[ref3] Arık M. B. (2003). Futbolun medyadaki temsilinde erkek egemen söylem. Kocaeli Üniversitesi İletişim Fakültesi Araştırma Dergisi 4, 65–82.

[ref4] Bozlu B. (2021). Türkiye’de Kadin Futbolcular: Kadin ve Erkek Futbolcularin Karşilaştirmali Ilişkisel Analizi (Yüksek Lisans Tezi). Antalya: Akdeniz Üniversitesi, Sosyal Bilimler Enstitüsü.

[ref5] Braun V. Clarke V. (2019). Reflecting on reflexive thematic analysis. Qual. Res. Sport Exerc. Health 11, 589–597. doi: 10.1080/2159676X.2019.1628806

[ref6] Braun V. Clarke V. (2021). Thematic Analysis: A Practical guide. London, United Kingdom: Sage.

[ref7] Bruce T. (2016). New rules for new times: sportswomen and media representation in the third wave. Sex Roles 74, 361–376. doi: 10.1007/s11199-015-0497-6

[ref8] Bruening J. E. Dixon M. A. (2008). Situating work-family negotiations within a life course perspective: insights on the gendered experiences of NCAA division I head coaching mothers. Sex Roles 58, 10–23. doi: 10.1007/s11199-007-9350-x

[ref9] Cin H. (2022). Türkiye’de ve Almanya’da kadın futbolcu olmak: Kadın futbolcuların yaşantılarının incelenmesi (Yayımlanmamış Yüksek Lisans Tezi). Ankara: Ankara Üniversitesi, Sağlık Bilimler Enstitüsü, Spor Bilimleri Anabilim Dalı.

[ref10] Cleland J. Doidge M. Millward P. Widdop P. (2020). Football, Gender and Fandom in Europe. London: Routledge.

[ref11] Connell R. W. Messerschmidt J. W. (2005). Hegemonic masculinity: rethinking the concept. Gender Soc. 19, 829–859. doi: 10.1177/0891243205278639

[ref12] Cooky C. Messner M. A. (2018). No Slam Dunk: Gender, Sport and the Unevenness of Social Change. New Brunswick: Rutgers University Press.

[ref13] Cooky C. Messner M. A. Musto M. (2015). “It’s dude time!”: a quarter century of excluding women’s sports in televised news and highlight shows. Commun. Sport 3, 261–287. doi: 10.1177/2167479515588761

[ref14] Creswell J. W. Poth C. N. (2018). Qualitative Inquiry and Research Design. 4th Edn Thousand Oaks, CA, United States: Sage.

[ref15] Culvin A. (2021). Football as work: the lived realities of professional women footballers in England. Manag. Sport Leis. 28:1959384. doi: 10.1080/23750472.2021.1959384

[ref16] Fasting K. Pfister G. (2000). Female and male coaches in the eyes of female elite soccer players. Eur. Phys. Educ. Rev. 6, 91–110. doi: 10.1177/1356336X000061007

[ref17] Fielding-Lloyd B. Meân L. (2011). “I don’t think I can catch it”: women, football and the media. Soccer Soc. 12, 365–379. doi: 10.1080/14660970.2011.568104

[ref18] FIFA (2020). Women’s football strategy Fédération Internationale de football association. Zurich, Switzerland: FIFA.

[ref19] FIFA (2022). Women’s Football Benchmark Report 2022 Fédération Internationale de Football Association. Zurich, Switzerland: FIFA.

[ref20] FIFA (2023). Setting the Pace: FIFA Benchmarking Report on Women’s Football. Zurich, Switzerland: FIFA.

[ref21] Fink J. S. (2015). Female athletes, women’s sport, and the sport media commercial complex: have we really “come a long way, baby”? Sport Manag. Rev. 18, 331–342. doi: 10.1016/j.smr.2014.05.001

[ref22] Galli N. Gonzalez S. P. (2015). Psychological resilience in sport: a review of the literature and implications for research and practice. Int. J. Sport Exerc. Psychol. 13, 243–257. doi: 10.1080/1612197X.2014.946947

[ref23] Gubby L. Martin S. (2024). Sexism, abuse and threatening behaviour: experiences of women football referees in amateur and semi-professional men’s football in the UK. Sport Educ. Soc. 30:2324376. doi: 10.1080/13573322.2024.2324376

[ref24] Gültekin G. (2021). Kadın futbolunun Türkiye’deki gelişimi ve medyada temsili. Spor Bilimleri Dergisi 32, 145–160.

[ref25] Hargreaves J. (1994). Sporting Females: Critical Issues in the History and Sociology of Women’s Sports. London: Routledge.

[ref26] Kavasoğlu İ. (2021). “Your hair or the team!” body politics and possibilities of resistance in women’s football in Turkey. Fe Dergi, 13, 38–52. doi: 10.46655/federgi.1067184

[ref27] Koca C. Bulgu N. (2005). Media portrayal of women’s sports in Turkey. Int. Rev. Sociol. Sport 40, 443–455. doi: 10.1177/1012690205060095

[ref28] LaVoi N. M. (2016). “A framework to understand experiences of women coaches around the globe,” in Women in Sports Coaching. In N. M. LaVoi (Ed.), Women in Sports Coaching. (New York, NY, United States: Routledge).

[ref29] Malterud K. Siersma V. D. Guassora A. D. (2016). Sample size in qualitative interview studies: guided by information power. Qual. Health Res. 26, 1753–1760. doi: 10.1177/1049732315617444, 26613970

[ref30] Messner M. A. (2002). Taking the Field: Women, Men, and Sports. Minneapolis: University of Minnesota Press.

[ref31] Moustakas C. (1994). Phenomenological Research Methods. Thousand Oaks: Sage Publications.

[ref32] Ntoumanis N. Ng J. Y. Y. Prestwich A. Quested E. Hancox J. E. Thøgersen-Ntoumani C. (2021). A meta-analysis of self-determination theory-informed intervention studies in the health domain: effects on motivation, health behavior, physical, and psychological health. Health Psychol. Rev. 15, 214–244. doi: 10.1080/17437199.2020.1718529, 31983293

[ref33] Öztürk P. (2017). Kadın futbolcuların futbol alanındaki deneyimler. Ankara: Hacettepe Üniversitesi Sağlık Bilimler Enstitüsü Spor Bilimler ve Teknolojisi Programı Doktora Tezi.

[ref34] Öztürk A. (2019). Türkiye’de kadın futbolunun yapısal sorunları ve medya görünürlüğü. Spor ve Toplum Araştırmaları Dergisi 2, 55–74.

[ref35] Öztürk P. (2019). Türkiye’de kadın futbolunun görünürlüğü: Medya ve toplumsal cinsiyet bağlamında bir inceleme. Toplum ve Bilim 146, 85–110.

[ref36] Öztürk P. Koca C. (2018). Futbolda kadınlar: Bir sosyal alan olarak kadın futbol takımının analizi. Türkiye Klinikleri Spor Bilimleri Dergisi 10, 150–163.

[ref37] Öztürk P. Koca C. (2022). Kadın futbolunda profesyonelleşme sürecinde medya ve sponsorluk ilişkileri. Spor Bilimleri Dergisi 33, 245–262.

[ref38] Patton M. Q. (2015). Qualitative Research and Evaluation Methods. 4th Edn Thousand Oaks: Sage Publications.

[ref39] Pfister G. (2010). Women in sport—gender relations and future perspectives. Sport Soc. 13, 234–248. doi: 10.1080/17430430903522954

[ref40] Pfister G. (2015). Assessing the sociology of sport: on women and football. Int. Rev. Sociol. Sport 50, 563–569. doi: 10.1177/1012690214566644

[ref41] Pope S. (2011). Like pulling down Durham cathedral and building a brothel: women as “new consumer” fans? Int. Rev. Sociol. Sport 46, 471–487. doi: 10.1177/1012690210384652

[ref42] Ryan R. M. Deci E. L. (2017). Self-Determination Theory: Basic Psychological Needs in Motivation, Development, and Wellness. New York, NY: Guilford Press.

[ref43] Ryan R. M. Deci E. L. (2020). Intrinsic and extrinsic motivation from a self-determination theory perspective. Contemp. Educ. Psychol. 61:101860. doi: 10.1016/j.cedpsych.2020.101860

[ref44] Sarkar M. Fletcher D. (2014). Ordinary magic, extraordinary performance: psychological resilience and thriving in high achievers. Sport Exerc. Perform. Psychol. 3, 46–60. doi: 10.1037/spy0000003

[ref45] Scraton S. Flintoff A. (2013). Gender, Sport and Physical Activity: A socio-Cultural Study. London: Routledge.

[ref46] Toffoletti K. Palmer C. (2017). New approaches for studies of Muslim women and sport. Int. Rev. Sociol. Sport 52, 146–163. doi: 10.1177/1012690215589326

[ref47] Weinberg R. S. Gould D. (2019). Foundations of Sport and Exercise Psychology. 7th Edn Champaign: Human Kinetics.

[ref48] Williams J. (2003). A Game for Rough Girls? A History of Women’s Football in Britain. London: Routledge.

[ref49] Zarrett N. Cooky C. Veliz P. T. (2019). Coaching Through a Gender Lens: Maximizing Girls’ Play and Potential. New York, NY: Women’s Sports Foundation.

