# Correction: Cleats on, battle real: sustainability and gender equality in women's football

**DOI:** 10.3389/fpsyg.2026.1868620

**Published:** 2026-05-14

**Authors:** Serda Ornek, Murat Yalcin Besiktas, Mustafa Serdar Terekli, Mert Erkan, Nalan Aksakal

**Affiliations:** 1 Faculty of Sport Sciences, Department of Sport Management, Fenerbahce University, Istanbul, Türkiye; 2 Faculty of Sport Sciences, Department of Coach Education, Fenerbahce University, Istanbul, Türkiye; 3 Department of Physical Education and Sports Teaching, Faculty of Sport Sciences, Fenerbahce University, Istanbul, Türkiye; 4 Faculty of Sport Sciences, Department of Physical Education, Eskisehir Technical University, Eskisehir, Türkiye

**Keywords:** gender equality, policy development, sport sustainability, structural barriers, women's football

Affiliation “Department of Physical Education and Sports Teaching, Faculty of Sport Sciences, Fenerbahce University, Istanbul, Türkiye” was erroneously given as “Faculty of Sport Sciences, Department of Recreation, Eskisehir Technical University, Eskisehir, Türkiye” for author Mustafa Serdar Terekli.

The original version of this article has been updated.

